# Beyond passive exposure: active engagement and green behavior amplify urban wellbeing

**DOI:** 10.3389/fpubh.2026.1797797

**Published:** 2026-04-07

**Authors:** Ning Chen, Yifan Kang

**Affiliations:** 1 Institute of Ecology and Sustainable Development, Shanghai Academy of Social Science, Shanghai, China; 2 School of Public Finance and Taxation, Shandong University of Finance and Economics, Jinan, China

**Keywords:** environmental satisfaction, multi-theory framework, pro-environmental behavior, SEM, wellbeing

## Abstract

Environmental conditions are increasingly recognized as important determinants of population health and wellbeing. However, the mechanisms linking environmental experiences to wellbeing remain insufficiently understood. Prior research rarely distinguishes between active environmental engagement and subjective environmental appraisal. It also typically examines these pathways in isolation. This study addresses this gap by using a pathway-specific multi-theory framework. We integrate Social Cognitive Theory, Attention Restoration Theory, and Social Exchange Theory in a single structural model. Using structural equation modeling and survey data from Shanghai residents, we examine three pathways linking environmental engagement, satisfaction, and green behavior to wellbeing. The results show that environmental engagement, satisfaction, and green behavior each have significant positive associations with wellbeing. In addition, green behavior partially mediates the relationship between environmental engagement and wellbeing. This indicates that the health-related benefits of engagement arise both directly and indirectly through behavioral translation. The findings contribute to the literature by providing simultaneous empirical evidence for distinct behavioral and affective pathways. We quantify their relative magnitudes and show that about two-fifths of the wellbeing benefits associated with environmental engagement are transmitted through green behavior. From a public health perspective, the results suggest that urban health promotion should move beyond improving environmental quality alone. Efforts should also foster residents’ active participation in environmental action. A dual-track strategy that combines lowering barriers to civic engagement and supporting sustained private-sphere green practices may generate broader wellbeing benefits in urban settings.

## 1 Introduction

Public health and environmental sustainability stand as two of the most critical global challenges of the 21st century (1). The United Nations’ 2030 Agenda for Sustainable Development, with its 17 Sustainable Development Goals (SDGs), sets out an integrated blueprint for improving both population health and environmental quality. Yet recent assessments suggest substantial shortfalls, with a sizeable share of SDGs, particularly those related to health and environmental conditions, stagnating or even regressing (2). This pattern highlights a fundamental interdependence: environmental degradation increasingly threatens human health and wellbeing, while human production and consumption activities accelerate ecological decline (3–5). Addressing these intertwined challenges therefore requires a clearer understanding of how individuals interact with their surrounding environment and how such interactions translate into wellbeing outcomes (6, 7).

A growing body of research has established that contact with natural environments is associated with improved physical health and psychological wellbeing (8–11), and can also foster pro-environmental outcomes (12–14). Meta-analytic evidence further indicates that nature connectedness, which refers to individuals’ subjective sense of relational ties to the natural world, is positively correlated with eudaimonic wellbeing and pro-environmental actions (15–18). However, much of the literature implicitly emphasizes benefits derived from passive exposure (i.e., being in or near nature), and comparatively less attention has been paid to the mechanisms through which active engagement and subjective appraisal transform environmental conditions into sustained behavioral change and durable wellbeing improvements (19–21).

### 1.1 Literature review and research gaps

Despite substantial progress, key gaps remain in disentangling the distinct pathways linking environmental factors to wellbeing. Prior work often examines cognitive or affective constructs [such as environmental awareness (22, 23), environmental perception (24–26), and nature connectedness (5, 27)] alongside behavioral outcomes, but typically in isolation rather than within a unified, mechanism-focused framework (28, 29). This fragmented approach makes it difficult to determine whether wellbeing gains arise primarily from active behavioral participation or from affective appraisal of one’s surroundings.

Central to this discussion is the role of subjective environmental appraisals. Environmental perception, defined as individuals’ subjective assessments of local environmental quality, including air, green spaces, and cleanliness, has been shown to predict enhanced wellbeing, in some cases more strongly than objective environmental measures (30–34). This suggests that how residents evaluate their environment may matter as much as the environment itself, pointing to a direct affective pathway from satisfaction to wellbeing. Notably, such subjective evaluations can also extend beyond wellbeing to shape behavioral outcomes. In the Chinese context, Lin et al. (2025) demonstrated that residents’ satisfaction with environmental policy instruments positively influenced their willingness to adopt low-carbon behaviors, with place attachment serving as a partial mediator (35), while Lin et al. (2024) further showed that different types of energy policies exerted differential effects on low-carbon consumption and travel behaviors (36). These findings reinforce the broader point that subjective appraisals constitute a potent mechanism linking environmental conditions to both affective and behavioral responses, yet the specific pathway from active environmental engagement through green behavior to wellbeing has received limited attention.

Despite these insights, the literature has not sufficiently clarified whether wellbeing gains arise primarily from active, public-sphere environmental engagement (e.g., civic reporting or volunteering) or from affective appraisals of local environmental quality (i.e., environmental satisfaction), nor whether the benefits of engagement accrue directly or require translation into repeated, private-sphere green practices (22, 23, 37, 38). Most studies rely on linear regression or examine single pathways, which cannot disentangle these competing mechanisms (22, 23, 39). Traditional unified theoretical frameworks may oversimplify these multifaceted dynamics, prompting calls for pathway-specific theorization to elucidate distinct mechanisms (24–26). Accordingly, this study focuses on separating and quantifying the contributions of a behavioral pathway via environmental engagement, an affective pathway via environmental satisfaction, and a behavioral-translation mechanism in which green behavior partially transmits the effect of engagement on wellbeing.

### 1.2 The current study

Building on the gaps identified above, this study addresses three research questions:

- RQ1: Do environmental engagement and environmental satisfaction exert distinct effects on individual wellbeing through different psychological mechanisms?
- RQ2: Does green behavior mediate the relationship between environmental engagement and wellbeing, and if so, what proportion of the total effect operates through this behavioral-translation channel?
- RQ3: Are these pathway-specific relationships robust after controlling for individual-level demographics and district-level objective environmental conditions?

To address these questions, we employ structural equation modeling (SEM) with data from 978 Shanghai residents. Our empirical model focuses on four core constructs: environmental engagement (EE; public-sphere behavioral participation), environmental satisfaction (ES; affective appraisal of local environmental quality), green behavior (GB; private-sphere pro-environmental practices), and wellbeing (WB). We test three pathways grounded in distinct theoretical foundations: (1) EE → WB via Social Cognitive Theory, (2) ES → WB via Attention Restoration Theory, and (3) EE → GB → WB, where GB mediates the relationship between EE and WB, interpreted through Social Exchange Theory. SEM is particularly suited to this task, as it models latent constructs, distinguishes direct from indirect effects, and accounts for measurement error in interrelated psychological variables (40, 41). By simultaneously assessing behavioral and affective predictors within a single model while controlling for key covariates, this study allows us to quantify the relative effect sizes of our core constructs and to distinguish direct psychological pathways from behavioral mediation. In doing so, we extend prior conceptual models of nature’s benefits (19) toward planetary health perspectives (42), offering empirical validation of distinct mechanisms and informing environmental policy and wellbeing interventions.

The remainder of the paper is organized as follows. Section 2 develops the conceptual framework and hypotheses. Section 3 describes the research setting, data, and measures. Section 4 presents the empirical results, including measurement model assessment, structural model estimation, and mediation analysis. Section 5 discusses the findings and their implications for urban environmental policy and wellbeing interventions.

## 2 Conceptual framework and hypotheses development

This study employs a pathway-specific multi-theory framework in which each hypothesized pathway is grounded in the theory that best explains its underlying psychological mechanism, while all pathways are estimated simultaneously within a single structural model. This approach occupies a deliberate middle ground between two alternatives common in the literature. At one end, single-theory approaches apply one overarching theoretical lens to all environment–wellbeing pathways, thereby obscuring mechanisms that are psychologically distinct. For example, environmental engagement is a deliberate, agency-driven process, whereas environmental satisfaction more often reflects automatic and restorative responses to lived surroundings. Treating these pathways as theoretically equivalent risks collapsing conceptually different processes into a single explanatory logic. At the other end, merely juxtaposing multiple theories acknowledge complexity but leaves them empirically disconnected. Without an integrated analytical framework, it becomes difficult to assess their relative explanatory power or specify how different pathways operate in relation to one another. Our framework avoids two limitations: by assigning Social Cognitive Theory to the behavioral pathway (EE → WB), Attention Restoration Theory to the affective pathway (ES → WB), and Social Exchange Theory to the mediation mechanism (EE → GB → WB), we preserve the explanatory precision of each theory while enabling direct, within-model comparison of their relative effect sizes. The rationale for this design rests on the recognition that behavioral engagement with environmental issues involves cognitive processes related to agency, efficacy, and goal achievement, while affective environmental experiences involve restorative processes related to stress reduction, attention restoration, and emotional regulation (43, 44). These fundamentally different psychological processes require distinct theoretical lenses to be properly understood and explained (19, 45). Accordingly, the following sections detail two primary pathways to wellbeing and the mediating role of green behavior.

### 2.1 Behavioral pathway: environmental engagement and wellbeing

Environmental engagement represents individuals’ active and sustained participation in public-sphere environmental activities. Social cognitive theory (SCT) provides an appropriate framework for understanding how environmental engagement influences wellbeing through mechanisms of agency, self-efficacy, and outcome expectations (42, 45). According to SCT, individuals derive satisfaction and wellbeing from exercising personal agency in valued domains (45, 46). Environmental engagement enables individuals to act as agents of change, providing opportunities to demonstrate competence and achieve meaningful goals. Research demonstrates that environmental activism and engagement are associated with increased life satisfaction, purpose in life, and psychological wellbeing (47, 48). The mechanism operates through enhanced self-efficacy beliefs, where successful environmental engagement strengthens individuals’ confidence in their ability to effect positive change, contributing to overall wellbeing (43, 49).

*Hypothesis 1 (H1): Environmental engagement positively influences wellbeing.*

### 2.2 Affective pathway: environmental satisfaction and wellbeing

Environmental satisfaction represents individuals’ subjective evaluation of environmental quality and their affective responses to environmental conditions. Attention restoration theory (ART) provides the theoretical foundation for understanding how environmental satisfaction influences wellbeing through restorative processes (44, 50). ART proposes that exposure to restorative environments, which is characterized by fascination, being away, extent, and compatibility helps restore depleted attentional resources and reduce mental fatigue (50, 51). Environmental satisfaction can be interpreted as an appraisal that one’s environment affords restorative qualities, which in turn facilitates stress recovery and attentional restoration (50, 51). Consistent with this appraisal–restoration pathway, individuals who report higher environmental satisfaction experience lower levels of stress, anxiety, and depression, along with increased positive affect and life satisfaction (52, 53). Unlike the behavioral pathway, the affective pathway operates through largely automatic and non-effortful processes that do not require active engagement (50, 54).

*Hypothesis 2 (H2): Environmental satisfaction positively influences wellbeing.*

### 2.3 Green behavior mediation: a social exchange framework

#### 2.3.1 Environmental engagement and Green behavior

Social cognitive theory (SCT) also helps explain the link between environmental engagement and specific green behaviors. Environmental engagement, as a form of public-sphere action, serves as a proximal predictor of private-sphere green behaviors through mechanisms of intention formation and behavioral self-regulation (43, 55). Research consistently demonstrates that individuals with higher environmental engagement are more likely to perform various green behaviors, from energy conservation to sustainable consumption (22, 38). This relationship is mediated by increased environmental self-efficacy and stronger behavioral intentions (49, 56).

*Hypothesis 3 (H3): Environmental engagement positively influences green behavior.*

#### 2.3.2 Green behavior and wellbeing

Social exchange theory (SET) provides a framework for understanding how green behavior contributes to wellbeing through reciprocal exchange processes with the environment and society (57, 58). Green behavior contributes to wellbeing through several exchange mechanisms: social approval and recognition from engaging in socially valued behaviors, a sense of contribution to collective environmental goods, and reciprocal benefits from environmental protection (59, 60). Empirical evidence shows that individuals who engage in pro-environmental behaviors report higher levels of life satisfaction, positive affect, and psychological wellbeing (61, 62). The SET framework suggests that green behavior creates a form of environmental social capital whereby individuals receive psychological returns on their environmental investments (59, 60).

*Hypothesis 4 (H4): Green behavior positively influences wellbeing.*

#### 2.3.3 The mediating role of green behavior

Building on the preceding links, social exchange theory (SET) also provides a theoretical grounding for understanding green behavior as a mediator between environmental engagement and wellbeing (57, 58). Environmental engagement creates the cognitive and motivational foundation for green behavior, which then generates the reciprocal benefits that enhance wellbeing. Evidence indicates that stronger environmental commitment and interconnectedness are associated with higher engagement in pro-environmental actions (63). Contextual environmental conditions also shape both wellbeing and pro-environmental behavior (64). Together, these findings support a sequential process from engagement to behavior to wellbeing benefits (22). The mediation mechanism suggests that the wellbeing benefits of environmental engagement may not be fully realized without translation into actual green behavior (59, 61).

Establishing whether this mediation exists, and quantifying its magnitude, is important for both theoretical and practical reasons. From a theoretical standpoint, the question of whether environmental engagement enhances wellbeing primarily through direct psychological rewards (such as heightened self-efficacy and a sense of purpose) or whether these benefits require behavioral translation into concrete green practices remains unresolved in the literature (22, 38). If the indirect pathway through green behavior accounts for a substantial share of the total effect, it would suggest that engagement alone is a necessary but insufficient condition for maximizing wellbeing gains; the behavioral channel must also be activated. Conversely, if the direct effect dominates, it would imply that the cognitive and affective experience of participation is itself the primary source of wellbeing. From a practical standpoint, distinguishing between these two mechanisms has direct implications for intervention design. A predominantly direct effect would favor policies that lower barriers to civic environmental participation (e.g., community reporting platforms, volunteer programs), whereas a strong indirect effect would additionally call for interventions that facilitate the translation of engagement into sustained private-sphere practices (e.g., nudges for energy conservation, green commuting infrastructure) (43, 59). To adjudicate between these possibilities empirically, we employ a bootstrap-based mediation test and report the indirect effect, its bias-corrected confidence interval, and the variance accounted for (VAF) ratio, which quantifies the proportion of the total effect that operates through the behavioral channel (65).

*Hypothesis 5 (H5): Green behavior mediates the relationship between environmental engagement and wellbeing.*

**Figure 1:**
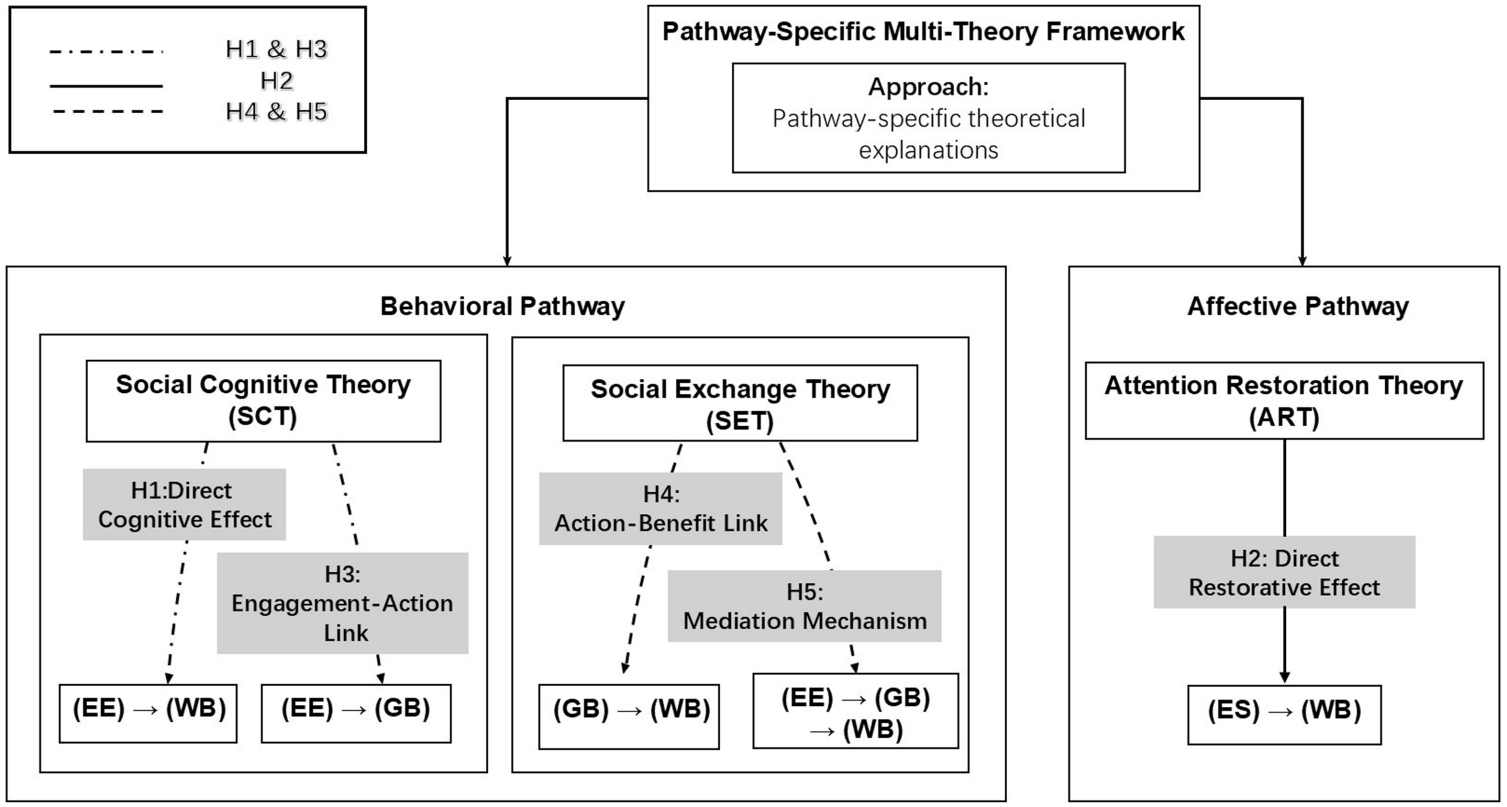
Conceptual framework and hypotheses development. SCT explains how engagement translates into action and wellbeing; SET explains how green behavior yields psychological returns; ART explains how environmental quality provides restorative benefits. **D**ash-dot lines for Social Cognitive Theory pathways (H1 and H3), solid lines for the Attention Restoration Theory pathway (H2), and dashed lines for Social Exchange Theory pathways (H4 and H5).

The conceptual framework and hypothesized relationships are presented in Figure 1.

## 3 Method

### 3.1 Research setting: Shanghai’s pro-environmental policy context

This study is situated in Shanghai, China’s largest economic hub, with a GDP of 5.39 trillion RMB in 2024 and a resident population of 24.8 million (66). As an international center in the fields of economy, finance, trade, shipping, and science and technology innovation, Shanghai has prioritized high-quality economic growth balanced with sustainable production and consumption. Since 2000, the city has implemented eight consecutive three-year environmental action plans, leading to significant improvements in ecological quality (67).

Shanghai is at the forefront of China’s efforts to promote sustainable urban living, with a robust policy framework emphasizing residents’ adoption of pro-environmental practices. Over the past decade, the municipal government has implemented comprehensive environmental-related regulations covering waste sorting, plastic reduction, green product consumption, and resource recycling. For instance, mandatory waste classification became a legal obligation in 2019; measures such as restrictions on disposable plastics, incentives for eco-friendly products, and promotion of green transportation have integrated pro-environmental principles into daily life. These policies have influenced residents’ behaviors and their attention to ecological issues and perceptions of the living environment. While existing research has often focused on behavioral outcomes, gaps remain in understanding how psychological factors (such as environmental awareness and perception) directly impact health in this policy-rich setting.

To address this gap, our study focuses on Shanghai as a living laboratory to investigate the direct effects of environmental engagement and satisfaction on wellbeing. Our sample data from the residents covers differences in age, education, occupation, income, and region. This approach allows us to examine key constructions within the city’s unique policy-driven context.

### 3.2 Data collection and sample

Data were collected between October and December 2022 using a self-administered online questionnaire hosted on Wenjuanxing (问卷星), a widely used survey platform in China. The questionnaire drew on the established theoretical frameworks outlined in Section 2 and on prior empirical studies to ensure content relevance and construct validity. Our sampling strategy used a multi-stage quota design covering four main dimensions: geographic district, gender, age, and education. We selected these to capture major axes of demographic and spatial heterogeneity in Shanghai’s urban population. Target benchmarks were from the 2020 Shanghai Seventh National Population Census. We implemented a dual-tier quota system to ensure structural diversity. Hard quotas were strictly enforced for geographic distribution and gender balance. The survey remained open until valid responses were obtained from all 16 districts, and a near-proportional gender ratio was achieved. Target quotas for age and education reflected proportional objectives aligned with census distribution. Quota fulfillment was monitored in real time through the Wenjuanxing platform’s dashboard. This enabled dynamic tracking of response counts across demographic cells. When specific cells neared their targets, survey access was limited to eligible respondents. Underrepresented cells led to targeted redistribution. For ages 56 and above, we did three rounds of booster recruitment via neighborhood committee WeChat groups. This channel has a much higher reach among older residents than general social media. Despite these interventions, the 56 + age group was still underrepresented (7.3% in the sample versus 23.4% in the census). We attribute this to the ‘digital divide’ of mobile-based surveys, which lowers responsiveness among older cohorts (68). To address this demographic skew, we include age as a covariate in our structural model and conduct sensitivity analyses. This helps ensure the robustness of our core theoretical pathways. To ensure geographic and demographic diversity, survey links were distributed through community WeChat groups, residential neighborhood committees, and social media platforms, with distribution quotas set across all 16 administrative districts and key demographic segments (age, gender, education). Table 1 reports the distribution of respondents across Shanghai’s 16 administrative districts. The sample covers all districts of the municipality, including both core urban areas and outer suburban districts, enhancing the geographic representativeness of the survey.

**Table 1:** Geographic distribution of the sample across Shanghai’s administrative districts (*N* = 958).

District of residence	Frequency	Percent (%)	Cumulative (%)
Pudong New Area	177	18.48	18.48
Huangpu District	60	6.26	24.74
Xuhui District	105	10.96	35.70
Changning District	39	4.07	39.77
Jing’an District	49	5.11	44.89
Putuo District	61	6.37	51.25
Hongkou District	40	4.18	55.43
Yangpu District	36	3.76	59.19
Minhang District	52	5.43	64.61
Baoshan District	57	5.95	70.56
Jiading District	94	9.81	80.38
Jinshan District	25	2.61	82.99
Songjiang District	31	3.24	86.22
Qingpu District	36	3.76	89.98
Fengxian District	49	5.11	95.09
Chongming District	47	4.91	100.00
Total	958	100.00	

**Figure 2:**
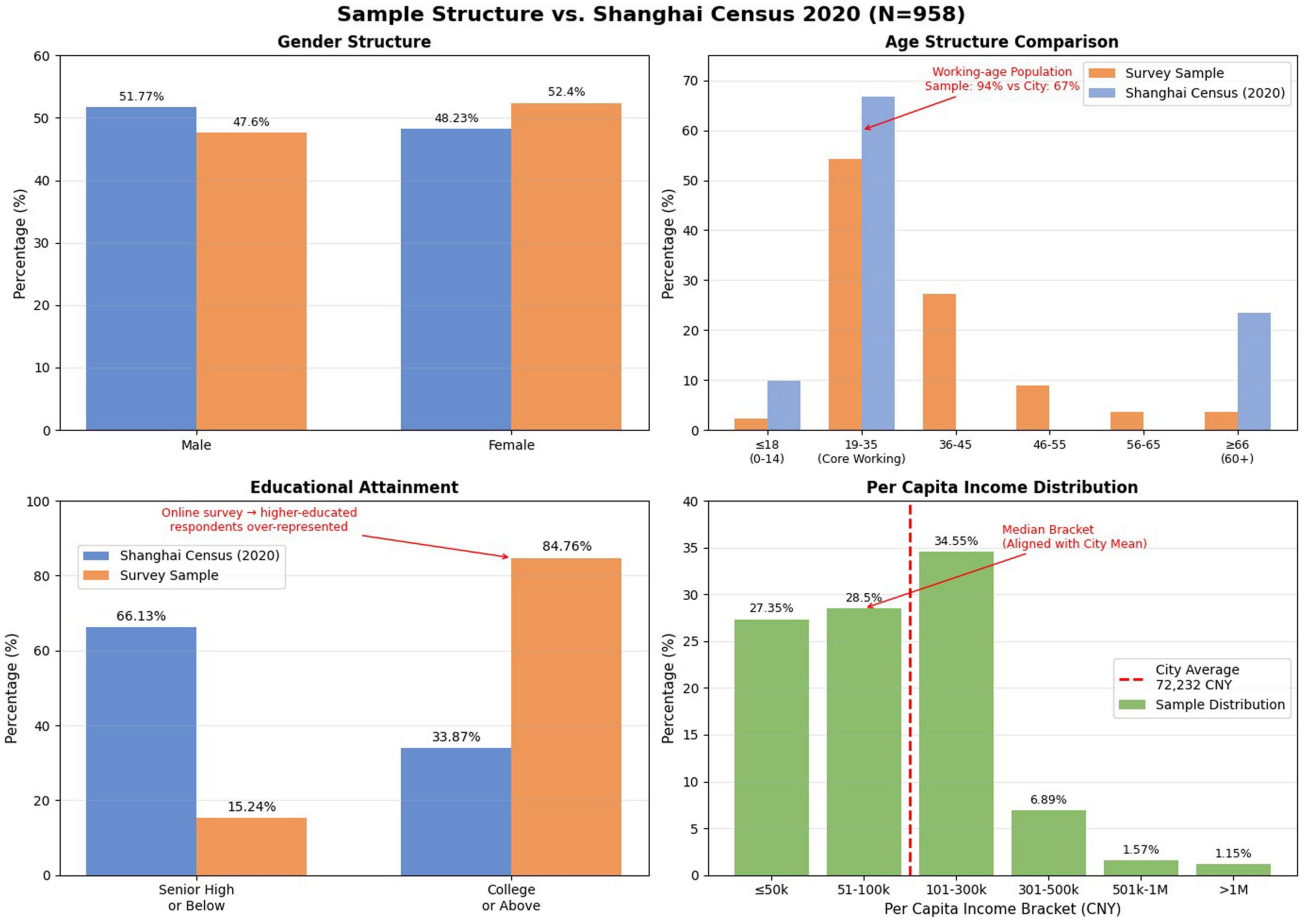
Comparison of sample demographic characteristics with Shanghai population benchmarks (*N* = 958). Census data are drawn from the Shanghai Seventh National Population Census (2020). Per capita disposable income benchmark (¥72,232) is from the Shanghai Municipal Bureau of Statistics (2022). “College or above” includes associate, bachelor, and postgraduate degrees.

Of the 1,000 questionnaires distributed, 978 were returned (response rate: 97.8%). After removing incomplete and low-quality submissions, 958 valid responses were retained (effective rate: 97.9%). The sample comprises 456 males and 502 females, aged 16 to 75, spanning various educational levels, occupations, income brackets, and all administrative districts of Shanghai. Figure 2 compares the sample’s demographic profile with population-level benchmarks from the Shanghai Seventh National Population Census (2020) and the Shanghai Statistical Yearbook (2022).

The sample is approximately gender-balanced (female 52.4%, male 47.6%), closely mirroring the census distribution (female 48.2%, male 51.8%). The age profile is concentrated in the working-age range: 90.4% of respondents are aged 19–55, compared with 66.8% of the census population aged 15–59, while respondents aged 56 and above account for 7.3% versus 23.4% (aged 60+) in the census. This pattern reflects the digital-access requirements of online surveys, which systematically underrepresent older cohorts (68). Educational attainment in the sample is substantially higher than in the general population: 84.8% of respondents hold a college degree or above, compared with approximately 33.9% in the census. The gap is attributable to two reinforcing factors: the younger age composition of the sample, given that college attainment rates among younger Shanghai cohorts far exceed the population average, and the self-selection inherent in web-based recruitment.

The sample spans the full range of per capita income brackets. The three largest groups are ≤50,000 CNY (27.4%), 51,000–100,000 CNY (28.5%), and 101,000–300,000 CNY (34.6%). Shanghai’s per capita disposable income was ¥72,232 in 2020 (69). The sample median falls within the 51,000–100,000 CNY bracket, indicating that the sample is not disproportionately skewed toward high-income residents; a substantial share of respondents (particularly younger adults in early career stages) report incomes below the city average.

The online sampling method produces a sample that is younger and more educated than the general Shanghai population, a pattern common to web-based survey research in urban China. This demographic profile is, however, well-suited to the present study, which examines environmental engagement and green behavior among urban residents who are active participants in civic and environmental life. To account for objective place-based differences, the structural model controls for three district-level environmental indicators (PM2.5, green space ratio, coastline distance; see Section 3.3.2). Although individual-level demographics were not entered as covariates in the SEM, the demographic composition of the sample is reported transparently above to allow readers to assess external validity.

### 3.3 Measures

The survey assessed four main constructs along with control variables at both the individual and district levels, encompassing demographics and objective environmental factors.

#### 3.3.1 Core constructs

All primary items except self-rated health status used a 5-point Likert scale ranging from 1 (“Rarely”) to 5 (“Always”), where higher scores indicated greater environmental engagement, more frequent green behavior, and higher environmental satisfaction. Self-rated health employed the same 5-point format but with anchors from 1 (“Very poor”) to 5 (“Very good”), where higher scores reflected better health status.

- Environmental Engagement (EE): Defined as civic actions in the public sphere aimed at promoting collective environmental wellbeing, consistent with Stern’s (2000) concept of public-sphere pro-environmental behaviors. This construct reflects a sense of environmental citizenship and was measured with two high-involvement indicators: (1) active intervention (e.g., “How often do you intervene or report behavior that harms the ecological environment?”) and (2) organized participation (e.g., “How often do you participate in environmental protection volunteer activities?”). These two indicators were selected to represent the two core dimensions of public-sphere pro-environmental behavior identified by Stern (2000): spontaneous civic action and organized collective participation.
- Green Behavior (GB): Operationalized as private-sphere practices intended to reduce personal environmental impact. Following the principle that measurement should focus on high-impact behaviors (38), we selected two indicators highly relevant to the Chinese urban context: (1) household energy conservation (setting air conditioning to 26 °C or higher) and (2) green commuting (prioritizing walking, cycling, or public transport). These two high-impact behaviors were selected for their direct policy relevance in the Shanghai urban context and to minimize respondent burden in a multi-construct survey instrument.
- Environmental Satisfaction (ES): Conceptualized as a resident’s subjective and affective assessment of their local physical environment. It was operationalized as a formative construct, consistent with prior multifaceted environmental assessments (70). Questions included items such as: “How satisfied are you with community environmental sanitation?” “How satisfied are you with park green spaces near your residence?” and “How satisfied are you with local air quality and water quality?” It is formed by four distinct indicators reflecting satisfaction with: (1) sanitation, (2) green spaces, (3) air quality, and (4) water quality. As these indicators are considered distinct causes that collectively define the construct, high intercorrelations are not expected.
- Wellbeing (WB): Defined as an integrative formative construct that expands beyond the mere absence of disease to include holistic functioning (71, 72). We contend that a comprehensive measure of health must encompass both subjective perceptions and restorative behaviors. It is formed by two complementary indicators: (1) self-rated health, capturing a holistic perception of one’s physical and mental state (73), and (2) frequency of visiting green spaces, which serves as a key restorative health behavior linked to stress reduction and attentional recovery (50, 74).

#### 3.3.2 Control variables

The survey collected individual-level demographic information, including age, gender, education, occupation, marital status, income, and membership in environmental organizations, to characterize the sample composition (see Section 3.2 and Figure 2). At the modeling stage, three district-level environmental indicators were included as control variables in the SEM to account for objective environmental conditions that may confound the relationships among the core constructors. Prior research has established that objective environmental conditions shape both residents’ subjective environmental appraisals and their wellbeing outcomes independently of individual-level characteristics (30, 31, 34). The three indicators, drawn from the Shanghai Statistical Yearbook, each represent a distinct dimension of objective environmental quality: (1) annual average PM2.5 concentration (μg/m^3^), capturing ambient air quality (4, 64); (2) ratio of park and green space (%), capturing urban green infrastructure accessibility (9, 51); and (3) distance to the nearest coastline (km), calculated using ArcGIS based on district centroids and the city’s shoreline, capturing proximity to natural landscape resources (53). As respondents reported only their district of residence, these indicators were controlled for at the district level. By including these controls, the model provides a partial robustness check against confounding from differences in objective environmental endowments across districts, though it does not fully disentangle district-level effects from the subjective and behavioral processes of interest.

## 4 Data analysis results

### 4.1 Measurement model assessment

This study employed Partial Least Squares Structural Equation Modeling (PLS-SEM) as the primary analytical tool, suitable for exploratory theory development, predictive analysis, complex models, formative constructs, smaller samples from limited populations, non-normal distributions, and when latent scores are needed for further analysis (65, 75, 76). PLS-SEM was chosen to build an integrated model examining the relationships among environmental engagement (EE), green behavior (GB), environmental satisfaction (ES), and wellbeing (WB). For significance testing, bootstrapping with 10,000 resamples was applied to ensure stable parameter estimates (65, 77).

#### 4.1.1 Reliability and convergent validity

To establish the convergent validity of the measurement model, we assessed the internal consistency, composite reliability (CR), and average variance extracted (AVE) for each construct. As shown in Table 2, factor loadings ranged from 0.621 to 0.909, with most exceeding the 0.70 benchmark, indicating good item convergence. Cronbach’s alpha values were as follows: EE (0.761), GB (0.664), ES (0.906), WB (0.786). Whereas ES and WB exceeded the 0.70 threshold, GB was slightly below, suggesting moderate reliability. Composite reliability (CR) values were: EE (0.627), GB (0.519), ES (0.707), WB (0.669), with ES exceeding 0.70 while others were lower, particularly GB. However, AVE values all exceeded 0.50 (EE: 0.770; GB: 0.679; ES: 0.906; WB: 0.799), supporting overall convergent validity despite some CR and alpha limitations, which are acceptable in exploratory PLS-SEM contexts (78). This provides a reasonable foundation for the latent constructs.

**Table 2:** Reliability and convergent validity of constructs.

Constructs	ITEMS	Factor loading	Cronbach’s alpha	Composite reliability (CR)	Average variance extracted (AVE)
EE	EE 1	0.760	0.761	0.627	0.770
	EE 2	0.822			
GB	GB1	0.808	0.664	0.519	0.679
	GB2	0.621			
ES	ES1	0.836	0.906	0.707	0.906
	ES2	0.836			
	ES3	0.862			
	ES4	0.830			
WB	WB 1	0.909	0.786	0.669	0.799
	WB 2	0.715			

EE = environmental engagement; GB = green behavior; ES = environmental satisfaction; WB = wellbeing. (1–5 Linkert).

#### 4.1.2 Discriminant validity

Discriminant validity was assessed using the Fornell-Larcker criterion, as presented in Table 3. The results demonstrate that the square root of the average variance extracted (AVE) for each construct exceeded its correlations with all other constructs, confirming adequate discriminant validity. For instance, the square root of AVE for Environmental Engagement (EE) was 0.792, which is substantially higher than its correlations with Green Behavior (GB) at 0.556, Environmental Satisfaction (ES) at 0.506, and Wellbeing (WB) at 0.502. Similarly, all other constructs met this criterion. These findings indicate that the constructs possess good discriminant validity, ensuring the independence and accuracy of each latent variable in the measurement model (78).

**Table 3:** Fornell-Larcker criterion.

Constructs	EE	GB	ES	WB
EE	0.792			
GB	0.556	0.720		
ES	0.506	0.411	0.841	
WB	0.502	0.364	0.426	0.818

The diagonal elements (highlighted values) in the correlation matrix represent the square root of AVE for each construct. EE = environmental engagement; GB = green behavior; ES = environmental satisfaction; WB = wellbeing.

### 4.2 Structural model results

#### 4.2.1 Direct effects

**Figure 3:**
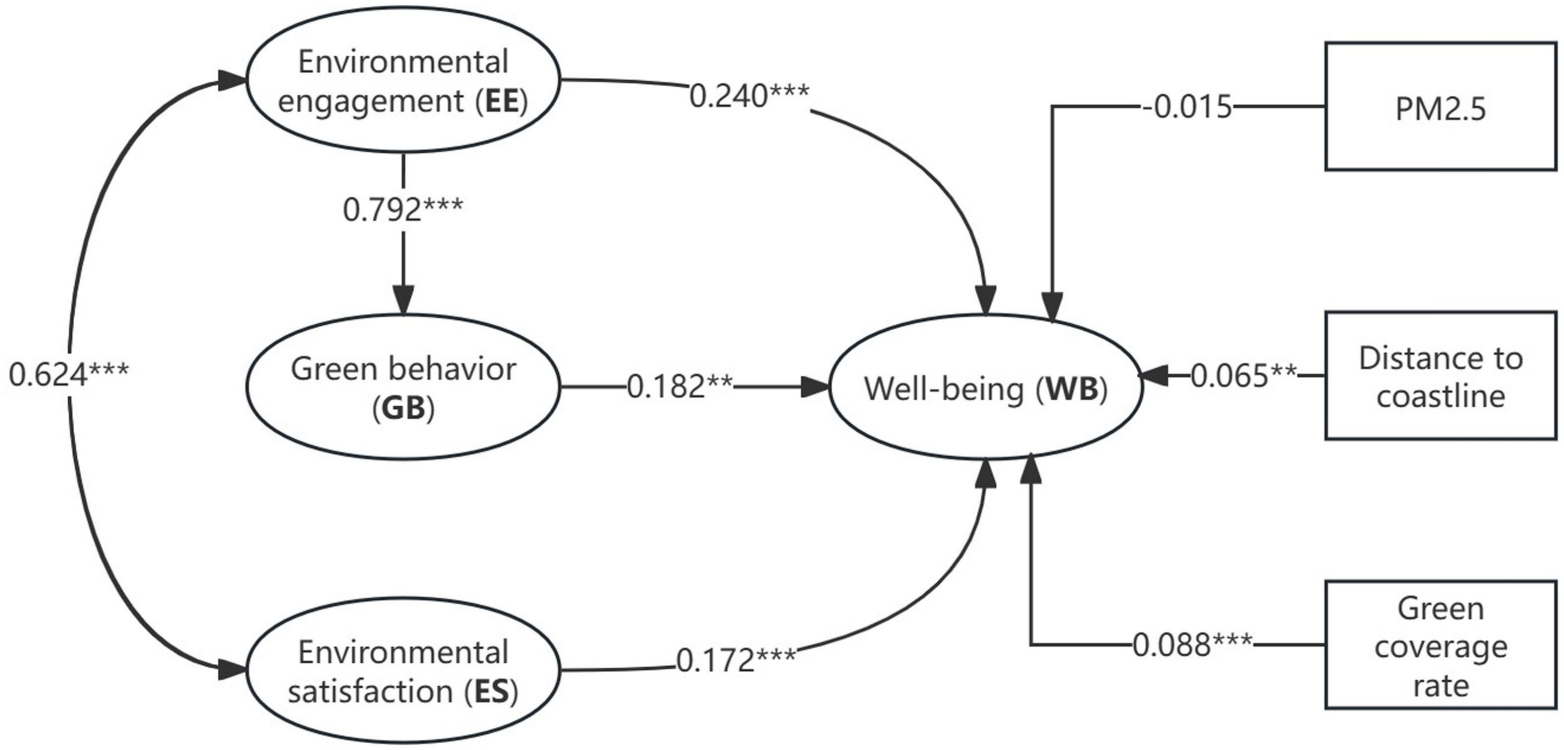
SEM of pathway analysis. Environmental engagement; GB = green behavior; ES = environmental satisfaction; WB = wellbeing. PM2.5, distance to coastline, and green coverage rate were included as district-level control variables. *, **, and *** denote statistical significance at the 10, 5, and 1% level, respectively.

In SEM, the path structure among constructs constitutes the structural model. Table 4 and Figure 3 present the path coefficients, t-values, significance levels, and hypothesis testing results for this inner model, respectively. Given the nested structure of the data, we estimated the model using cluster-robust standard errors at the district level. In Table 4, these are reported as “Robust SE.” A full multilevel SEM was not estimated because the number of second-level units (16 districts) is below the level generally considered adequate for stable higher-level estimation (79). We therefore adopted cluster-robust standard errors to adjust for within-district dependence, while recognizing that inference based on a limited number of clusters should be interpreted with caution (80). The data analysis results demonstrate that all proposed hypotheses (H1-H4) in this study are supported. These results provide strong empirical support for the proposed theoretical framework, as all hypothesized direct effects were found to be statistically significant. The details are as follows:

**Table 4:** Model path coefficient diagram.

Hypothesis	Relationship	Path coefficient (Standardized)	Robust SE	*T*-value	*p*-value	Decision
H1	EE → WB	0.240***	0.088	2.720	0.006	Supported
H2	ES → WB	0.172***	0.042	4.110	0.000	Supported
H3	GB → WB	0.182**	0.087	2.100	0.036	Supported
H4	EE → GB	0.792***	0.033	23.680	0.000	Supported
—	PM2.5 → WB	−0.015	0.030	−0.500	0.619	Control variable
—	Distance to coastline → WB	0.065**	0.028	2.290	0.022	Control variable
—	Green coverage rate → WB	0.088***	0.030	2.960	0.003	Control variable

EE = environmental engagement; GB = green behavior; ES = environmental satisfaction; WB = wellbeing. PM2.5, distance to coastline, and green coverage rate were included as district-level control variables. Standard errors are cluster-robust at the district level to adjust for within-district dependence. *, **, and *** denote statistical significance at the 10, 5, and 1% level, respectively.

Specifically, environmental engagement had a significant positive effect on wellbeing (H1: *β* = 0.240, z = 2.72, *p* = 0.006), supporting Social Cognitive Theory’s proposition that engagement enhances wellbeing through agency and self-efficacy. Environmental satisfaction also exerted a significant positive effect on wellbeing (H2: *β* = 0.172, z = 4.11, *p* < 0.001), consistent with Attention Restoration Theory. Green behavior positively predicted wellbeing as well (H3: *β* = 0.182, *z* = 2.10, *p* = 0.036), in line with Social Exchange Theory’s notion of reciprocal psychological benefits. Finally, environmental engagement showed a strong and significant positive effect on green behavior (H4: *β* = 0.792, z = 23.68, *p* < 0.001), confirming the motivational pathway from engagement to behavioral expression.

Collectively, these results validate the hypothesized direct relationships in the model. The path coefficients, ranging from moderate to strong, confirm the theoretical foundations underlying the environmental engagement-wellbeing nexus. To assess the robustness of the core pathways, three district-level environmental indicators were included as observed exogenous controls with direct paths to wellbeing (See details in Table 4 and Figure 3). The green space ratio had a significant positive effect on wellbeing (*β* = 0.088, SE = 0.030, *z* = 2.96, *p* = 0.003, 95% CI [0.030, 0.146]), consistent with the restorative benefits of urban green infrastructure. Distance to the nearest coastline was also positively associated with wellbeing (*β* = 0.065, SE = 0.028, *z* = 2.29, *p* = 0.022, 95% CI [0.009, 0.120]). Although the blue space literature generally links coastal proximity to health benefits in European recreational-coast settings (81, 82), Shanghai’s coastline is dominated by industrial ports and logistics infrastructure rather than accessible waterfronts, and greater coastline distance effectively proxies for residence in the city’s well-serviced core urban districts. Annual average PM2.5 concentration showed a non-significant effect (*β* = −0.015, SE = 0.030, z = −0.50, *p* = 0.619, 95% CI [−0.073, 0.043]), suggesting that once subjective environmental satisfaction is accounted for, district-level air quality variation does not independently predict wellbeing (83). Importantly, after the inclusion of these controls, all hypothesized direct paths (H1–H4) remained positive and statistically significant. Although some coefficients changed in magnitude, the direction, significance, and substantive interpretation of the core relationships remained unchanged, supporting the robustness of the theoretical model.

To examine whether the age-skewed sample affected the structural results, we conducted a sensitivity analysis by re-estimating the model with six age-group dummy variables. The results were substantively unchanged: all hypothesized paths remained statistically significant, and the standardized coefficients were nearly identical to those in the baseline model. For example, the effect of green behavior on health status changed only from 0.1815 to 0.1855, the effect of environmental awareness on health status from 0.2397 to 0.2374, and the effect of environmental awareness on green behavior from 0.7920 to 0.7922. These results suggest that the main findings are robust to the sample’s age composition.

#### 4.2.2 Mediation analysis

To test H5, we assessed the indirect effect of environmental engagement (EE) on wellbeing (WB) through green behavior (GB) using the bootstrap method with 10,000 resamples (65). As shown in Table 5, the indirect effect was statistically significant (indirect effect = 0.116, SE = 0.027, *z* = 4.23, *p* < 0.001), with a 95% confidence interval of [0.062, 0.170], which does not include zero, providing robust evidence for mediation. The variance accounted for (VAF) was 40.96%, calculated as the ratio of the indirect effect to the total effect of EE on WB (0.116/0.284=0.410) . According to Hair et al. (2014), a VAF between 20 and 80% indicates partial mediation (65).

**Table 5:** Mediating effect test.

Hypothesis	Variable relationship	Mediated effect	VAF	Robust SE	*T*-value	*P*-value	95% Boot CI	Accept/Reject
H5	EE= > GB= > WB	0.116***	40.96%	0.027	4.23	<0.001	(0.062, 0.170)	Accept

EE = environmental engagement; GB = green behavior; ES = environmental satisfaction; WB = wellbeing. The percentile bootstrap method was employed for the analysis. Standard errors are cluster-robust at the district level to adjust for within-district dependence. *, **, and *** denote statistical significance at the 10, 5, and 1% level, respectively.

This result carries substantive implications. The finding that approximately two-fifths of the total effect of environmental engagement on wellbeing is transmitted through green behavior indicates that engagement yields wellbeing benefits through two concurrent channels: a direct psychological channel, reflecting the immediate cognitive and affective rewards of participation, consistent with Social Cognitive Theory, and an indirect behavioral channel, whereby engagement motivates private-sphere green practices that generate additional reciprocal wellbeing returns, consistent with Social Exchange Theory. The remaining effect operating directly suggests that the psychological experience of environmental participation is itself a potent source of wellbeing, independent of whether it translates into specific green actions. H5 is therefore supported, confirming partial mediation.

### 4.3 Model fit assessment results

The structural model’s fit was evaluated using multiple indices, indicating excellent alignment with the data: *χ*^2^(47) = 162.761 (*p* < 0.001); CFI = 0.970; TLI = 0.959; RMSEA = 0.051 (95% CI [0.042, 0.059], pclose = 0.428); SRMR = 0.047; CD = 0.976. While *χ*^2^ was significant which is likely due to the large sample size, other metrics exceeded benchmarks (e.g., CFI/TLI > 0.95; RMSEA < 0.06; SRMR < 0.05), confirming close fit (84). In addition, the goodness-of-fit (GOF) index of 0.456 further exceeds the 0.36 threshold for strong fit in PLS-SEM (85). These results affirm the model’s robustness and explanatory power.

The PLS-SEM analysis validated the model’s pathways, with strong convergent and discriminant validity despite minor reliability issues for GB. All hypotheses were supported, including partial mediation via GB. The excellent fit metrics provide a reliable empirical basis, deepening insights into environmental factors’ role in wellbeing and suggesting avenues for scale refinement and moderator exploration (86).

## 5 Discussion

This study examined how environmental factors relate to individual wellbeing within the highly structured, policy-driven setting of Shanghai. We used a multi-theory framework to disentangle the separate channels through which environmental engagement, satisfaction with one’s surroundings, and green behavior might influence wellbeing. Our results validate the proposed theoretical pathways and provide a more textured account of wellbeing, illustrating how distinct cognitive, affective, and behavioral processes operate within the context of Shanghai’s environmental-oriented policy background.

### 5.1 A case for theoretical specificity

The central contribution of our paper lies in the empirical argument for theoretical specificity. In a field where the trend has often been toward all-encompassing, unified models, our results make a case for a more disaggregated approach. The data show that distinct psychological mechanisms, each better explained by a separate theory, connect different environmental variables to wellbeing. For example, the direct, positive relationship between environmental satisfaction and wellbeing (*β* = 0.172) fits squarely within the predictions of Attention Restoration Theory, which argues that quality environments offer automatic, restorative benefits that buffer against mental fatigue (50). At the same time, the fact that environmental engagement independently predicts wellbeing resonates with a core tenet of Social Cognitive Theory: that exercising personal agency on issues of consequence fosters a sense of efficacy and self-worth (43).

To be precise about what we mean by a “multi-theory framework”: our approach is a pathway-specific theoretical architecture in which each structural pathway is matched to the theory whose mechanisms it most closely instantiates, and all pathways are then tested simultaneously within a single SEM. This is distinct from single-theory accounts that would, for example, invoke Social Cognitive Theory to explain both the engagement and satisfaction pathways, that is, an application that would strain the theory’s scope conditions, since SCT’s core constructs of agency and self-efficacy do not naturally account for the automatic, non-effortful restorative processes that characterize the satisfaction–wellbeing link (44, 50). It is equally distinct from *ad hoc* theory juxtaposition, where multiple theories are cited but never brought into empirical contact within a shared model. By embedding pathway-specific theories in a single structural equation model, we can directly compare the standardized effect sizes of the behavioral pathway (*β* = 0.240), the affective pathway (*β* = 0.172), and the mediation channel (indirect effect = 0.116). This provides quantitative evidence of the relative contribution of each theoretical mechanism, something that neither single-theory approaches nor simple theory juxtaposition can achieve. By treating these as separate, parallel pathways, we avoid the kind of theoretical oversimplification that can obscure important causal details (19). The analysis suggests that wellbeing in an urban context is supported by at least two independent pillars: the passive benefits drawn from a quality environment and the active benefits derived from cognitive and emotional investment in that environment.

This research also clarifies the role of mediation in the environment-wellbeing relationship. The finding that green behavior partially mediates the effect of environmental engagement (VAF = 40.96%) provides strong evidence for a dual-process model. It suggests that a person’s psychological orientation toward the environment yields wellbeing returns both directly, through immediate cognitive rewards, and indirectly, by prompting tangible actions that are themselves rewarding. This may help explain inconsistent findings in the literature. Where some studies find strong links between pro-environmental attitudes and wellbeing, others do not; our model suggests that the strength of this link may depend on whether and how behavior is included in the analysis. In showing that both the direct psychological pathway and the behaviorally-mediated one are significant, we affirm that while engagement is itself beneficial, its positive effects are amplified when it serves as a catalyst for action (83, 87). Importantly, the VAF of 40.96% points to a dual-channel interpretation with clear intervention implications. Because roughly three-fifths of the engagement–wellbeing effect is direct, policies that encourage civic environmental participation (such as community reporting platforms, environmental volunteering, and public awareness campaigns) can be expected to yield immediate psychological dividends even before any behavioral change occurs. At the same time, the remaining two-fifths transmitted through green behavior indicates that these dividends are meaningfully amplified when participation is accompanied by sustained private-sphere practices such as energy conservation and green commuting. This dual-channel finding argues against single-track interventions: programs that focus exclusively on promoting engagement without facilitating their translation into daily green habits will capture only a portion of the potential wellbeing gains, while programs that target behavior alone may lack the motivational foundation that engagement provides. A more effective strategy, therefore, is a coordinated “dual-track” approach that simultaneously lowers barriers to civic participation and provides the infrastructure, nudges, and feedback loops necessary to convert that participation into routine pro-environmental action.

Finally, our findings contribute to the application of Social Exchange Theory in this domain. The robust pathway from green behavior to wellbeing lends credence to the idea that pro-environmental actions are not simply altruistic sacrifices but are part of a reciprocal exchange that delivers psychological returns. Activities like conserving energy or choosing public transport can instill a sense of competence and social contribution, which Stern (2000) framed as a form of non-material benefit. This process can be understood as the accumulation of “environmental social capital,” where acting for the collective good reinforces one’s sense of purpose and connection, ultimately bolstering personal wellbeing.

### 5.2 A closer look at the mechanisms

#### 5.2.1 Unpacking the pathway mechanisms

A key advantage of our approach is that it allows us to inspect the character of each pathway. The Behavioral Pathway, grounded in Social Cognitive and Social Exchange Theories, reveals a two-part mechanism. The strong coefficient linking environmental engagement to green behavior (*β* = 0.792) identifies engagement as a potent motivational force. This has immediate relevance for intervention design, suggesting that policies aimed at moving people from passive awareness to active concern are a powerful lever for changing behavior (43). The subsequent positive link to wellbeing confirms that these actions complete a rewarding psychological cycle. This pathway is thus not a simple chain, but a dynamic process involving direct cognitive rewards from engagement and secondary, reinforcing rewards from the resulting behavior.

In contrast, the Affective Pathway appears to operate through a more passive, automatic process, as predicted by Attention Restoration Theory (50). While the effect size is more modest (β = 0.159), the direct relationship between environmental satisfaction and wellbeing represents a separate and complementary source of welfare. This channel requires no conscious effort; instead, it captures the direct benefits that high-quality surroundings confer, such as reduced stress and renewed focus. This finding is important because it validates a two-pronged strategy for enhancing public wellbeing: one that fosters active citizen participation, and another that centers on the fundamental provision of quality public spaces.

The Mediation Pathway then tells a sequential story. The partial mediation result suggests a layered process where individuals first reap immediate psychological benefits from being engaged, and these benefits are later supplemented when that engagement is converted into action (88).

#### 5.2.2 Interpreting findings within Shanghai’s policy context

These mechanistic insights gain additional significance when interpreted within the specific policy environment in which our data were collected. As described in Section 3.1, Shanghai has implemented one of China’s most comprehensive environmental policy frameworks, including mandatory domestic waste sorting since July 2019, restrictions on single-use plastics, incentives for green product consumption, and sustained investment in public transit and cycling infrastructure. Our survey was administered in late 2022, approximately 3 years after the most prominent of these regulations took effect. The green behaviors captured by our instrument, household energy conservation and green commuting, were therefore practiced within a regulatory environment that actively promotes, and in some cases legally mandates, pro-environmental conduct.

This policy context raises a theoretically important question: do the wellbeing benefits of green behavior differ depending on whether the behavior is policy-driven or voluntary? Self-Determination Theory (SDT) offers a useful framework for thinking through this issue (45). SDT posits that behaviors yield the greatest psychological returns when they are experienced as self-determined rather than externally controlled. In the environmental domain, this implies that green behaviors undertaken out of personal conviction should generate stronger wellbeing gains than identical behaviors performed solely to comply with regulations (59). If this reasoning held strictly, one might expect the GB → WB pathway to be attenuated in a highly regulated setting like Shanghai, where at least some green behaviors are mandated rather than freely chosen.

Yet our results tell a different story. The GB → WB pathway is both statistically significant and substantively meaningful (*β* = 0.182, *p* = 0.036), and green behavior serves as a significant partial mediator of the engagement–wellbeing relationship (VAF = 40.96%). One plausible interpretation, consistent with SDT’s concept of internalization, is that Shanghai residents have progressively integrated policy-mandated behaviors into their personal value systems over the 3 years since implementation. SDT describes internalization as a process whereby externally regulated behaviors are gradually transformed into identified or integrated regulations, forms of motivation that are experienced as autonomous and that satisfy basic psychological needs (45). Shanghai’s sustained policy implementation, combined with extensive public education campaigns, community-level enforcement, and visible environmental improvements, may have facilitated this process, such that behaviors initially adopted under regulatory pressure are now experienced as personally meaningful. This interpretation aligns with recent evidence from Shanghai showing a gradual shift from externally driven compliance to internally motivated waste sorting behavior (89).

This contextual reading also enriches our understanding of the mediation result. The strong EE → GB coefficient (*β* = 0.792) suggests that environmental engagement is a potent catalyst for green behavior. In Shanghai’s policy environment, this catalytic effect may be amplified because the city’s well-developed environmental infrastructure (ubiquitous recycling stations, extensive public transit networks, community-based environmental programs) lowers the practical barriers to translating engagement into action. Shanghai’s policy framework may therefore function as a structural facilitator, strengthening the behavioral-translation channel identified in our mediation analysis and helping explain why approximately two-fifths of the engagement–wellbeing effect operates through green behavior.

An important caveat is that our cross-sectional design and our aggregate green behavior measure do not allow us to directly distinguish between policy-mandated and voluntary behaviors, nor can we observe the internalization process longitudinally. It remains possible that wellbeing returns differ across specific behavior types; for instance, waste sorting required by local regulation may generate weaker autonomous motivation than voluntary green commuting, and the aggregate GB → WB coefficient may mask such heterogeneity. We return to this limitation below and identify it as a priority for future research.

### 5.3 Practical and policy relevance

These findings have clear implications for practice and policy. First, this study confirms that environmental engagement (EE) enhances wellbeing (WB), suggesting the need to lower participation barriers, simplify procedures, and make EE a routine part of daily life. To this end, a tiered and differentiated EE platform should be established. At the municipal level, resources from environmental organizations could be integrated into a unified EE information platform that disseminates opportunities for volunteering, outreach, education, and public welfare projects. This would enable one-stop access to participation opportunities. At the local level, participation options should be tailored to different groups. For example, adolescents could engage in practice-based learning, working-age adults could take on flexible volunteer roles, and older adults could support outreach and communication. Such programs would encourage residents to participate in environmental activities within their communities.

Second, this study shows that satisfaction with the ecological environment is a key factor in residents’ WB, highlighting the importance of improving environmental quality, optimizing green space provision, and enhancing residents’ positive experiences. Policy efforts should focus on aspects of the residential environment with low satisfaction, such as cooking fumes, noise, and construction waste. More research and more refined governance are needed in areas that remain weak points, such as ozone $\left(O_{3}\right)$ , volatile organic compounds (VOCs), and emerging pollutants. To expand green spaces with clear health benefits, efforts should be accelerated to develop urban parks, community green spaces, and waterfront greenways, so that residents can access green space within a 15-min walk.

Third, this study finds that green behavior (GB) serves as an important mediating pathway linking EE and WB, indicating the need to further improve incentive mechanisms in order to promote the broader adoption of GB among residents. Three policy recommendations follow. First, the existing carbon incentive mechanism should be further refined to strengthen the positive economic feedback associated with GB. In particular, the range of recognized carbon-reduction scenarios could be gradually expanded to include high-frequency household GB, such as waste sorting, the use of energy-efficient appliances, rooftop photovoltaic installation, and second-hand trading. The associated benefits could also be broadened to include public service benefits, cultural benefits, and health-related benefits, thereby enhancing both practical value and public appeal. Second, the provision of relevant products and infrastructure should be strengthened so that GB can become a normalized part of everyday life. For example, the wider deployment of smart recycling machines and intelligent waste collection facilities could increase residents’ willingness to participate; greater investment in the development and promotion of green products could improve the accessibility of green consumption; and further optimization of public transport networks, together with improvements in active mobility infrastructure, could help address commuting barriers to green travel. Third, symbolic incentives and social recognition should be reinforced to cultivate voluntary and internalized behavioral commitment. Continuous public communication and education on GB are needed, alongside greater efforts to identify connections between Shanghai’s local green culture and GB, so as to enhance residents’ emotional resonance and foster enduring green behavioral habits.

### 5.4 Limitations and future directions

This study is not without limitations. First, its cross-sectional design means we can identify correlations but cannot make definitive causal claims. Longitudinal data would allow tracking the temporal ordering of these variables and observing how these relationships unfold over time (31).

Second, our reliance on self-report measures carries the risk of common method bias. While statistical checks suggest this was not a major concern, self-reported pro-environmental behavior is susceptible to social desirability and recall biases that may inflate observed associations (39). Future work could produce more robust findings by incorporating objective data, such as smart meter energy readings or administrative data on park usage.

Third, environmental engagement and green behavior were each measured with two items. Although two-indicator constructs are viable in PLS-SEM, where constructs are estimated as composites rather than common factors (65), this parsimony limits the content coverage of both scales. For green behavior in particular, the relatively low loading of the green commuting indicator (0.621) suggests that commuting mode choice is influenced by contextual factors beyond environmental motivation (such as residential distance and transit accessibility) introducing construct-irrelevant variance. While the items were deliberately chosen for their policy relevance and behavioral specificity in urban China, and the AVE for both constructs exceeds the 0.50 threshold (78), future research should employ more comprehensive, validated multi-item scales (such as broader civic participation inventories and multi-domain green behavior batteries) to capture a fuller range of engagement and behavioral practices. Moreover, because our green behavior items do not distinguish between policy-mandated and voluntary actions, we cannot directly test whether the wellbeing returns of green behavior differ by motivational origin. Future research could employ longitudinal or quasi-experimental designs to track the internalization process predicted by Self-Determination Theory, and to quantify how the autonomy dimension of green behavior moderates its effects on wellbeing (for instance, comparing cohorts surveyed before and after the introduction of mandatory regulations).

A fourth methodological consideration concerns the nested structure of our data, in which 958 individual observations are distributed across 16 administrative districts. Although a full multilevel structural equation model (MSEM) represents the gold standard for disentangling individual-level and district-level effects, its estimation stability depends on the number of higher-level units. According to the guidelines of Maas and Hox (2005), stable estimation in MSEM typically requires at least 30 s-level units, a condition that is not met in our 16-district sample. To address potential within-district dependence while preserving model stability, we employ district-level cluster-robust standard errors, a widely used correction in the econometric literature (80). This approach produces more conservative inference by accounting for intra-cluster correlation, but it does not provide a full multilevel decomposition of district-level confounding. Therefore, the findings related to Research Question 3, which examine the robustness of pathways after controlling for district-level environmental indicators, should be interpreted as a partial robustness check rather than definitive evidence of district-level mechanisms. Future research using data from a larger number of geographic units would enable full MSEM estimation and a more rigorous separation of individual-level and district-level effects.

Future work could build on this foundation by: (1) using longitudinal or experimental designs to test the causal pathways proposed here; (2) pursuing cross-cultural validation to see if this framework holds in different institutional settings; and (3) investigating moderators, such as personality or income, that might influence whether individuals draw more wellbeing from the behavioral or the affective pathway.

## 6 Conclusion

Using survey data from 978 respondents in Shanghai as the empirical baseline, the coupling mechanism between pro-environmental behaviors and environmental satisfaction is examined in this paper from a pathway-specific multi-theory framework, in which distinct theories are matched to the specific psychological mechanisms underlying each pathway. Also, empirical scrutiny is applied to the separate behavioral and affective pathways, which provides granular evidence for addressing the “stagnating” progress of the UN Sustainable Development Goals regarding the health-environment nexus. According to the structural equation results, a dual-process effect on wellbeing exists, manifested by the fact that both the passive enjoyment of environmental quality (affective route) and active public-sphere participation (behavioral route) independently propel individual wellbeing. Notably, the validity of these distinct channels holds under strict model fit criteria, differentiating this study from traditional unified frameworks that often conflate these dynamics. As revealed by mechanistic analyses, green behavior functions as a pivotal partial mediator, effectively converting the cognitive investment of engagement into a reciprocal utility gain via Social Exchange theory. Neglecting this behavioral mediation channel would lead researchers and policymakers to underestimate the true wellbeing returns of environmental engagement by approximately two-fifths, as the indirect pathway through green behavior represents a substantial and statistically significant portion of the total effect. Consequently, the promotion of public health requires a symbiotic policy paradigm: optimizing environmental infrastructure while simultaneously triggering the agency of residents to maximize psychological returns.

## Data Availability

The raw data supporting the conclusions of this article will be made available by the authors, without undue reservation.
